# The UK Veterinary Immunological Toolbox Website: promoting vaccine research by facilitating communication and removing reagent barriers

**DOI:** 10.1111/imm.13227

**Published:** 2020-07-29

**Authors:** William Mwangi, Giuseppe Maccari, Jayne C. Hope, Gary Entrican, John A. Hammond

**Affiliations:** ^1^ The Pirbright Institute Woking UK; ^2^ Royal Free Hospital Anthony Nolan Research Institute London UK; ^3^ The Roslin Institute The University of Edinburgh Midlothian UK

**Keywords:** antibodies, comparative immunology, immunological reagents, veterinary vaccinology

## Abstract

Using the best animal models to study immune responses against specific pathogens or vaccines can dramatically accelerate our understanding. Veterinary species are well studied, particularly livestock, to reduce their disease burden. They have also proven to be powerful models, especially for zoonotic pathogens and novel vaccination strategies. A prerequisite for any model selection is having the right quality and range of species‐specific immunological reagents. To help promote the widest possible use of veterinary species, an open access website (https://www.immunologicaltoolbox.co.uk) has been created as a central community annotated hub for veterinary immunological reagents. The website is also the portal into services offered by the UK Immunological Toolbox project that includes antibody generation, sequencing and recombinant expression. The funding for this effort is linked into sustainable sources, but ultimate success relies on community engagement to continually increase the quality and quantity of information. It is hoped that as more users and reagent owners engage, it will become an essential resource for researchers, veterinarians and clinicians alike by removing barriers that prevent the use of the most informative animal models.

## Graphical abstract

A central community annotated database and website have been created to share reagent information and facilitate the study of veterinary species. It is hoped that with continued community engagement, https://www.immunologicaltoolbox.co.uk/ will promote the use of the most informative animal models, maximizing the enormous potential of comparative immunological studies.

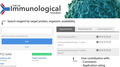

The potential of comparative immunology has never been greater. New high‐throughput technologies are becoming increasingly adaptable across species, creating enormous opportunities to push the utility and applicability of comparative research. Zoonotic infections continue to emerge, and novel vaccine design and delivery challenges often require large‐scale development and testing in animal models.^1^, ^2^ The continuous co‐evolution of host–pathogen relationships has driven the complexity and diversity of the innate and adaptive immune systems between different species.^3^ Several basic principles of immunobiology were established by exploiting opportunities that different animals offer. Sheep were used as an experimental system to study lymphocyte recirculation in studies initiated over 50 years ago. These studies made a seminal contribution to current immunotherapies involving adoptive transfer of T cells in humans.^4^ B‐cell immunology originates from studies on the Bursa of Fabricius in chickens, a species that has also helped to establish the principles of major histocompatibility complex class I peptide selection and loading.^5^ Using the right animal models can dramatically improve the quality and relevance of the data to reduce the development time of therapeutics. The pig is a natural mixing vessel of influenza viruses and is emerging as an important model for disease and vaccination.^6^ Calves have proven essential to understand the mechanisms of immunity and pathogenesis of human and bovine respiratory syncytial virus, proving essential to evaluate human vaccine candidates.^7^ Many species, particularly dogs, have underpinned our fundamental understanding of oncogenesis, including virally induced cancers and subsequent vaccines and immunotherapies.^8^, ^9^ However, the power of any given model is dependent on the availability and quality of the tools and reagents available.^10^

Although there are commonalities in immune system structure and function, at the molecular level there is often low homology between orthologues across species, such as cytokine/cytokine receptor interactions.^11^ Consequently, immunological reagents tend to exhibit low species cross‐reactivity, a feature that is often overlooked when moving from human and mouse studies into novel species. The immunological toolboxes for laboratory mice and humans are extensive, but cannot necessarily be applied to livestock, companion animals or wildlife species. For example, only 55% of anti‐human antibodies tested on pig blood cells gave consistent binding, with a subset showing novel cellular distribution compared with humans, with clear gaps remaining in what are clearly fundamental immunological pathways.^12^, ^13^ This creates a barrier to understanding host–pathogen interactions in those species and identifying the mechanisms of protective immunity that can underpin strategic vaccine design. Improving the immunological toolbox across species will enable comparative immunological studies and expedite the development of strategies for disease control.

Until the late 1980s, research in veterinary species was fundamental to our basic understanding of mammalian immune system function and development.^14^ The technological landmark of monoclonal antibody production in the 1980s drove a massive acceleration in tools and technologies to investigate the immune system at ever finer resolution.^15^ Murine immunology emerged and remains at the centre of fundamental discoveries that underpinned key immunological paradigms that still stand today. However, monoclonal antibody specificity often precludes reactivity with the equivalent protein orthologues in other species, and inevitably reagents for veterinary species lagged behind. The global effort to equip the veterinary immunological toolbox at the molecular level began in the 1980s with a notable drive to produce monoclonal antibodies that could identify lymphoid cell subsets. These efforts were largely focused, but not exclusively, around livestock and poultry to help design and improve disease control measures in key food‐producing species.^16^

The investment in veterinary antibodies has largely relied on limited‐term funding opportunities, spread across different species, countries and often diverse disease problems. This inevitably creates challenges in the coordination and knowledge exchange of past and future efforts that short‐term funding is unable to consolidate. The United Kingdom Research and Innovation – Biotechnology and Biological Sciences Research Council (UKRI‐BBSRC) Veterinary Vaccinology Strategy 2015–2020 highlighted this funding issue alongside the acknowledgement that that there are generic vaccinology research gaps that would benefit from coordinated research efforts with immunology, technology and immunological reagents being the three main long‐term research challenges globally and in the UK.^17^

The Pirbright Institute (TPI) and The Roslin Institute (TRI) are strategically funded UKRI‐BBSRC Institutes with a remit to undertake and promote research driving improvements in farmed animal health and productivity. Both Institutes have a strong track record in developing veterinary immune reagents, which has placed them in an ideal position to link immunological toolbox activities with their core strategic funding. This is a new funding model providing a greater level of financial sustainability that has led to the creation of the UK Immunological Toolbox project. Over the last 2 years TPI and TRI have coordinated and consolidated their veterinary toolbox activities and engaged as widely as possible with the research community on how to promote veterinary immunology research.

A community‐focused reagent database and website portal with consolidated information was considered by far the most immediate need of the community. It was also recognized that community buy‐in was key to make such a website accurate and current. To address this need the UK Immunological Toolbox website (https://www.immunologicaltoolbox.co.uk) was recently released and provides a manually curated and centralized resource of information on over 1600 antibodies and recombinant proteins from nine species, including livestock, companion animals and fish. These reagents include those available from commercial companies, institutional collections and individual laboratories. We estimate that we describe over half of the characterized antibodies held by the community globally; with many more partners committed to providing reagent information soon.

The website has been designed to present basic information (clone name, isotype, target, cross‐reactivity, etc.) alongside as much evidence as possible that promotes their use, such as references, methods and images. This includes the capacity for registered users to ‘rate’ reagents based on their own experiences in different applications and to provide comments and supporting evidence in almost any format. All comments and information are linked to the registered users that submitted the data, and are passed to the curation team to ensure basic quality. This rating feature is intended to accelerate research by only sourcing and trialling antibodies that are likely to work in any particular application. As such, the reagent owner and/or supplier is prominently displayed alongside current availability. Where applicable, links to species‐specific websites and resources are provided, allowing a greater level of detail to avoid duplication or wasted effort.

Research community engagement is equally as important as sustainable funding. Increasing the quality and volume of data will secure the future of this database and website. An international panel of volunteers with a research background for species have been identified to help promote the website and curate the information. The submission feature is located on the homepage and the form is designed to make it easy for users to submit new reagents or information on existing reagents with automated fields where possible.

The current more secure funding allows planning for future developments. The underlying data structure and website are highly scalable and are already capable of hosting additional information such as expression patterns, interacting partners, functions, isoforms and epitopes. There is also the option for the cDNA and DNA information, from genome locations, single nucleotide polymorphisms, copy number variations, splice variants and polymerase chain reaction primers and probes, to be added in the future.

The website also acts as the portal to access other initiatives and services offered through the UK Immunological Toolbox project through online submission. The development of new reagents can be suggested based on individual and community need and reviewed by an expert steering committee. We have also started a major project to sequence all hybridoma cells held at both TPI and TRI, as well as those from collaborating institutes around the world as requested. The primary driver is to secure these important reagents for the future, while also allowing us to share reagents at the level of sequence or construct; where available recombinant versions of antibodies are listed. When combined with our panel of vectors that enable the expression of recombinant antibodies of multiple isotypes from mouse, cattle, pig and chicken, the utility of these veterinary antibodies is greatly increased.

This capability helped to attract The Livestock Antibody Hub (www.immunologicaltoolbox.co.uk/hub) to TPI, which is contributing to the capacity and capability to sequence single B cells from livestock species. In combination with the UK Immunological Toolbox expression system, this now allows the direct generation of monoclonal antibodies from host veterinary species. This creates exciting new opportunities for new pathogen and species reagents, exploiting the variable properties that antibodies from different species can offer, that can also be engineered to have altered functions in different host species.

There are conserved molecular structures, biochemical processes and functional pathways between humans, biomedical species, companion animals and veterinary species that enable the use of established *in vitro*/*in vivo*/*in ovo* model systems. However, differences between species may restrict the interpretation of the outcomes of such model systems. Understanding the individual species and comparative responses becomes particularly important when pathogens emerge. Studying the immune responses of these species and taking a One Health approach more generally (http://www.onehealthglobal.net/) can provide solutions to health problems that originate at the animal–human–ecosystem interface.^18^ The success of such approaches inevitably depends on the availability of well‐characterized reagents and established/readily accessible experimental methods across multiple species. The UK Immunological Toolbox project is part of this initiative working with partners across the globe. We hope that veterinary immunologists and clinicians alike use and engage with this resource to accelerate their own research, as well as benefitting others by providing information.

## Disclosures

The authors have no competing interests to declare.
